# Synthesis of Mono- and Di-Glucosides of Zearalenone and α-/β-Zearalenol by Recombinant Barley Glucosyltransferase *Hv*UGT14077

**DOI:** 10.3390/toxins9020058

**Published:** 2017-02-09

**Authors:** Herbert Michlmayr, Elisabeth Varga, Francesca Lupi, Alexandra Malachová, Christian Hametner, Franz Berthiller, Gerhard Adam

**Affiliations:** 1 Department of Applied Genetics and Cell Biology, University of Natural Resources and Life Sciences, Vienna (BOKU), Konrad Lorenz Straße 24, 3430 Tulln, Austria; gerhard.adam@boku.ac.at; 2 Department of Food Chemistry and Toxicology, University of Vienna, Währinger Straße 38, 1090 Vienna, Austria; 3 Center for Analytical Chemistry and Christian Doppler Laboratory for Mycotoxin Metabolism, Department of Agrobiotechnology (IFA-Tulln), BOKU, Konrad Lorenz Straße 20, 3430 Tulln, Austria; elisabeth.varga@boku.ac.at (E.V.); francesca.lupi@unifg.it (F.L.); alexandra.malachova@boku.ac.at (A.M.); franz.berthiller@boku.ac.at (F.B.); 4 Dipartimento di Scienze Agrarie, degli Alimenti e dell’Ambiente, Università degli Studi di Foggia, Via-Napoli 25, 71122 Foggia, Italy; 5 Institute of Applied Synthetic Chemistry, Vienna University of Technology, Getreidemarkt 9/163, 1060 Vienna, Austria; christian.hametner@tuwien.ac.at

**Keywords:** zearalenone-14-glucoside, zearalenone-16-glucoside, masked mycotoxin, glycosylation, sucrose synthase, secondary metabolite

## Abstract

Zearalenone (ZEN) is an estrogenic mycotoxin occurring in *Fusarium*-infected cereals. Glucosylation is an important plant defense mechanism and generally reduces the acute toxicity of mycotoxins to humans and animals. Toxicological information about ZEN-glucosides is limited due to the unavailability of larger amounts required for animal studies. *Hv*UGT14077, a recently-validated ZEN-conjugating barley UDP-glucosyltransferase was expressed in *Escherichia coli*, affinity purified, and characterized. *Hv*UGT14077 possesses high affinity (*K*_m_ = 3 µM) and catalytic efficiency (*k*_cat_/*K*_m_ = 190 s^−1^·mM^−1^) with ZEN. It also efficiently glucosylates the phase-I ZEN-metabolites α-zearalenol and β-zearalenol, with *k*_cat_/*K*_m_ of 40 and 74 s^−1^·mM^−1^, respectively. *Hv*UGT14077 catalyzes *O*-glucosylation at C-14 and C-16 with preference of 14-glucoside synthesis. Furthermore, relatively slow consecutive formation of 14,16-di-glucosides was observed; their structures were tentatively identified by mass spectrometry and for ZEN-14,16-di-glucoside confirmed by nuclear magnetic resonance spectroscopy. Recombinant *Hv*UGT14077 allowed efficient preparative synthesis of ZEN-glucosides, yielding about 90% ZEN-14-glucoside and 10% ZEN-16-glucoside. The yield of ZEN-16-glucoside could be increased to 85% by co-incubation with a β-glucosidase highly selective for ZEN-14-glucoside. Depletion of the co-substrate UDP-glucose was counteracted by a sucrose synthase based regeneration system. This strategy could also be of interest to increase the yield of minor glucosides synthesized by other glucosyltransferases.

## 1. Introduction

Fungal infestation of crop plants is a world-wide threat to food safety and security. *Fusarium* species primarily infect small cereal crops and maize and produce several toxic metabolites during infection of their host plants [1]. Trichothecene-class mycotoxins (esp. deoxynivalenol, DON) and the resorcylic acid lactone zearalenone (ZEN) are the most problematic *Fusarium* metabolites [1,2]. DON is a potent virulence factor of *Fusarium* spp. and, like other trichothecenes (e.g., nivalenol and T-2 toxin), highly toxic for humans and animals. ZEN possesses low acute toxicity, but it is known for its estrogenic activity causing hyperestrogenism syndromes, which can lead to infertility in farm animals [2,3]. ZEN mainly occurs in the late phases of *Fusarium* infections and is less frequently detected than DON. However, it can accumulate when harvesting is delayed due to cool and humid climate conditions [3,4]. Whether ZEN has a significant role in plant pathogenesis is still unclear. Unlike the trichothecene toxins, ZEN exhibits low apparent phytotoxicity [5] and several *Fusarium* strains deficient in polyketide synthase genes essential for ZEN synthesis did not show reduced pathogenicity on wheat and barley [6,7,8]. Nevertheless, transcriptome analysis of the model plant *Arabidopsis thaliana* revealed that stress response-related proteins are induced in response to ZEN treatment [9]. 

The cellular detoxification mechanisms of plants can cope with xenobiotics by chemical modifications (phases I and II) followed by compartmentation of the resulting metabolites (phase III) [10]. Phase I (activation) provides functional groups for conjugation in phase II, e.g., by hydrolysis or redox-reactions catalyzed by the cytochrome P-450 system, but this does not necessarily reduce toxicity. In phase II, toxins and/or their phase I derivatives are conjugated by covalent linkage to hydrophilic molecules (e.g., glycosyl-, glutathione- and sulfate-residues, [11,12,13,14]). Glycosylation is a major route in phase II detoxification, but its importance for the cellular homeostasis of plants reaches far beyond defense. Physiological functions further encompass storage and transport of endogenous secondary metabolites and plant hormone regulation [15]. Glycosylation is catalyzed by nucleotide-sugar-dependent glycosyltransferases (GT) that are also referred to as Leloir enzymes [16]. Of particular importance are the UDP-sugar dependent GTs (UGT) assigned to family 1 (GT1) within the CAZy classification system of the glycosyltransferases [15,17]. Family 1 UGTs constitute a large gene family in plants that has undergone a significant expansion during the evolution of land plants [18]. For example, over 100 putative GT1 UGTs of *Arabidopsis thaliana* and about 180 of rice (*Oryza sativa*) have been identified through analysis of the plant secondary product glycosyltransferase (PSPG) motif, a highly conserved region among family 1 UGTs [15].

The importance of glycosylation in response to *Fusarium* infections was indicated by induction of several UGT genes in wheat [19,20,21,22]. Furthermore, the primary detoxification metabolite of DON, DON-3-glucoside has been detected in a wide range of cereals and cereal products with varying concentration levels [23]. To which extent glycosylation of DON is directly involved in the quantitative resistance of crop plants to *Fusarium* infections still needs clarification. Evidence that a major *Fusarium* resistance quantitative trait locus (QTL) is based on increased glycosylation of the virulence factor DON [24] was disputed [25]. Nevertheless, overexpression of a DON-conjugating glucosyltransferase clearly led to increased *Fusarium* resistance of transgenic wheat [26], strongly supporting the hypothesis that glycosylation can play an important role in *Fusarium* resistance. 

**Figure 1:**
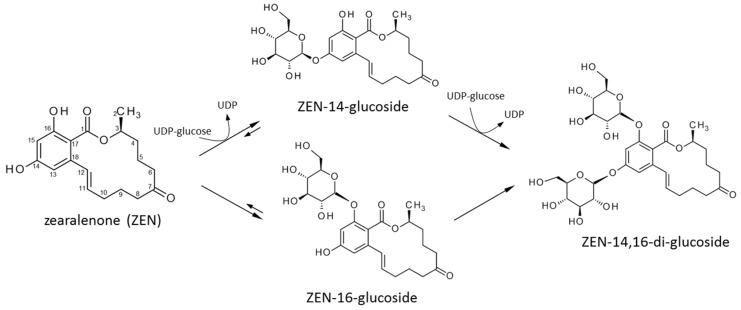
Scheme of reactions catalyzed by *Hv*UGT14077 with zearalenone and the glucosyl-donor UDP-glucose. *Hv*UGT14077 catalyzes the reversible conversion of zearalenone to zearalenone-14-glucoside and zearalenone-16-glucoside. Both metabolites are further conjugated to zearalenone-14,16-diglucoside. The phase I ZEN-metabolites α- and β-zearalenol are conjugated to their respective 14-/16-mono- and di-glucosides analogously.

ZEN is a target for plant defense systems as well. This was first indicated by the observation that maize suspension cultures convert ZEN to the phase I metabolites α- and β-zearalenol (αZEL/βZEL) and to their glucose conjugates [27,28]. ZEN-14-*O*-glucoside (ZEN-14-G, formerly designated ZEN-4-*O*-glucoside; see reference [29] for the numbering/nomenclature used here) is of particular relevance as a ZEN detoxification product. It was shown that glucosylation prevents the binding of ZEN to the human estrogen receptor [30]. An array of 17 different metabolites has been identified upon treatment of the model plant *Arabidopsis* *thaliana* with ZEN [31]. Apart from αZEL/βZEL, these included the 14-*O*-glucosides of ZEN and its phase I metabolites. More complex conjugates, such as di-hexosides, pentosyl-hexosides, and malonyl-glucosides of ZEN, αZEL, and βZEL were also observed. Elucidating the nature of the responsible mechanisms, in particular of those leading to glycosylation of ZEN and its phase I metabolites is promising to increase our understanding of crop plant response to pathogens. The first ZEN-conjugating UGT (*At*UGT73C6) producing ZEN-14-G was identified in *Arabidopsis* [30]. A phylogenetically related [32] barley UGT (*Hv*UGT14077) was transcriptionally induced by *Fusarium* infection and DON application [33,34], but found to be incapable to glucosylate DON [35]. Heterologous expression in *Saccharomyces cerevisiae* revealed that *Hv*UGT14077 can convert ZEN to ZEN-14-G and the previously unknown metabolite zearalenone-16-*O*-glucoside (ZEN-16-G, Figure 1) [36]. 

This study reports the biochemical characterization of recombinant *Hv*UGT14077 aiming to provide a functional profile of this defense-related enzyme. Substrate specificities and the reaction kinetics with ZEN and its phase I metabolites (αZEL/βZEL) were determined, leading to the identification of 14,16-di-glucosides as previously unknown plant detoxification metabolites of ZEN, αZEL, and βZEL. We further investigated preparative synthesis of ZEN-glucosides by *Hv*UGT14077 to generate sufficient amounts thereof for toxicological studies. A novel biocatalytic strategy to direct catalysis towards 16-glucoside synthesis was developed. 

## 2. Results

### 2.1. Biochemical Characteristics of HvUGT14077

**Figure 2:**
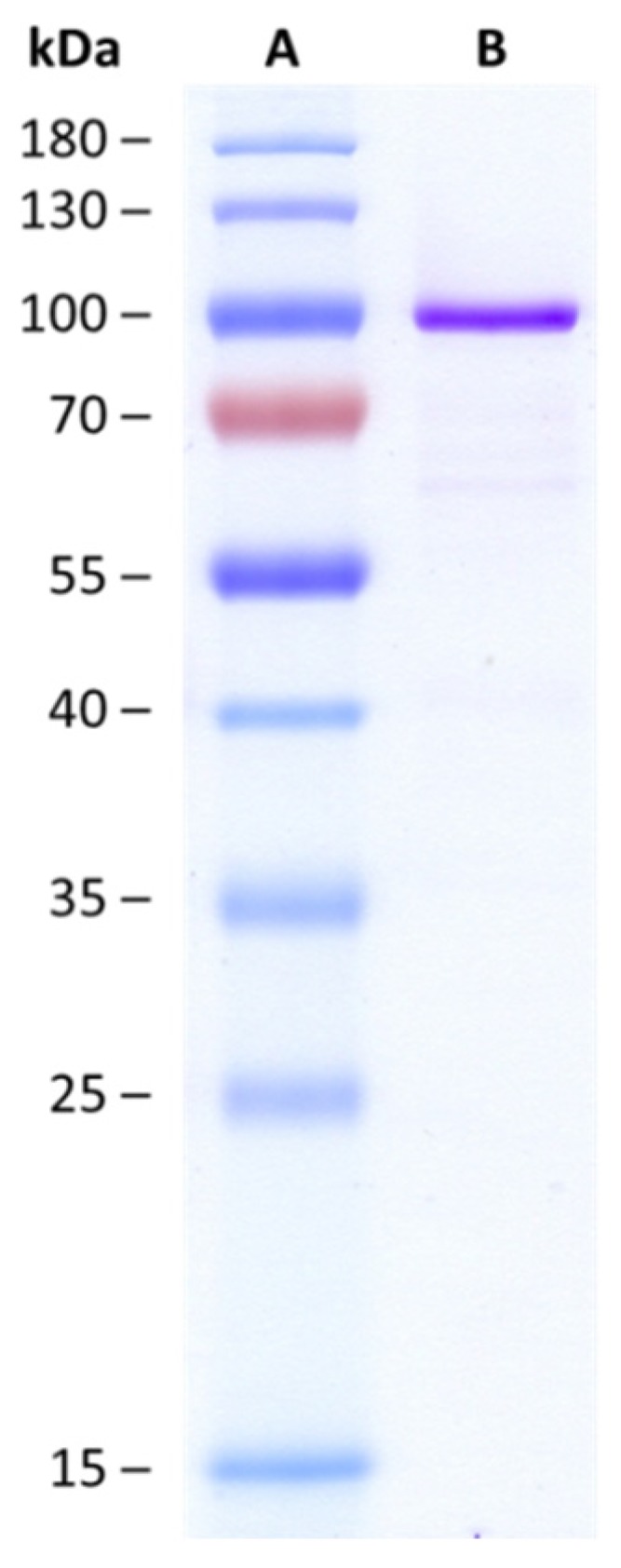
Sodium dodecyl sulfate polyacrylamide gel electrophoresis (SDS-PAGE). Lane A, PageRuler 10–180 kDa Prestained Protein Ladder (Thermo Scientific). Lane B, one-step-purified barley UDP-glucosyltransferase *Hv*UGT14077 expressed as fusion protein (nHIS_6_-MalE-TEV-*Hv*UGT14077) with a theoretical molecular mass of 97 kDa.

**Figure 3:**
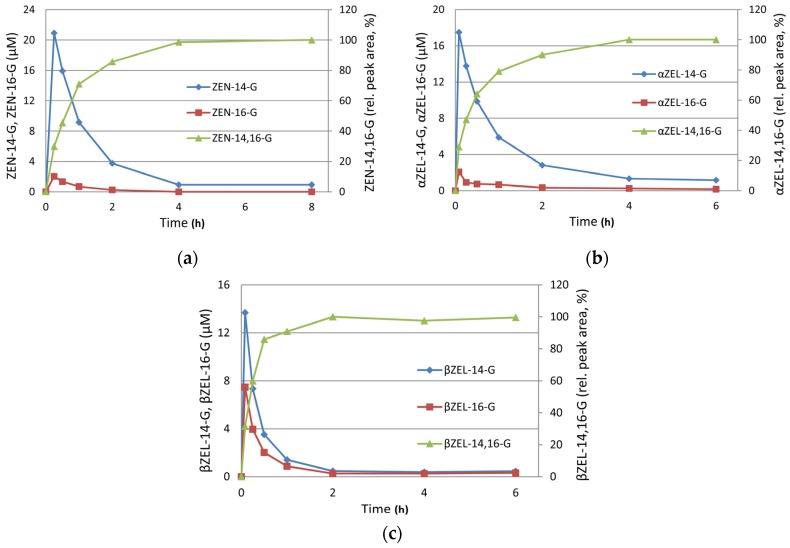
Time course of glucosylation of zearalenone (ZEN, (**a**)), α-zearalenol (αZEL, (**b**)) and β-zearalenol (βZEL, (**c**)) by *Hv*UGT14077 (2 mg·mL^−1^) with initial substrate concentrations of 25 µM and an excess of 10 mM UDP-glucose. The averages of duplicate determinations are shown.

*Hv*UGT14077 was expressed in *Escherichia coli* as a fusion protein with *N*-terminal His_6_- and maltose binding tag, followed by one-step metal ion affinity chromatography (IMAC) purification (Figure 2). In an initial experiment with 2 mg·mL^−1^ purified protein in the assay, ZEN (25 µM) was completely glucosylated within 15 min, yielding approximately 90% ZEN-14-G and 10% ZEN-16-G (Figure 3a, second time-point). With continued reaction the concentrations of both mono-glucosides decreased again and analysis by liquid chromatography coupled to tandem mass spectrometry (LC-MS/MS) revealed the formation of a third metabolite that, by its molecular mass, was tentatively identified as a putative ZEN-di-glucoside. A second peak of the same mass was not detected, supporting the hypothesis that both mono-glucosides were further converted into a ZEN-14,16-di-glucoside (ZEN-14,16-diG) rather than into individual ZEN-14- or ZEN-16-di-glucosides (e.g., gentiobiosides). These would have been formed by subsequent conjugation of a secondary glucosyl-residue to the primary glucose moiety. This was further supported by comparison of high-resolution mass spectrometry (HRMS) data with a ZEN-14-gentiobioside standard [37] which had a different retention time, 12.9 min compared to 10.3 min of the ZEN-di-glucoside. Furthermore, the structure of the purified ZEN-di-glucoside was confirmed by nuclear magnetic resonance (NMR) spectroscopy as ZEN-14,16-diG (see Section 2.3). 

*Hv*UGT14077 is also able to glucosylate the phase I metabolites αZEL and βZEL. Under identical conditions (2 mg·mL^−1^ *Hv*UGT14077, 25 µM substrate concentrations) as with ZEN, the reactions proceeded similar with rapid initial formation of the 14-, and 16-mono-glucosides and their further conjugation to 14,16-di-glucosides (Figure 3b,c). These metabolites were tentatively identified by high resolution tandem mass spectrometric measurements (MS/HRMS) (see below). 

In agreement with the previously reported inability to confer DON-resistance when the *Hv*UGT14077 gene was expressed in yeast [35], glucosylation of DON and nivalenol could not be detected.

Reaction rates of *Hv*UGT14077 were determined at 25 µM substrate and 10 mM co-factor concentration (Table 1). ZEN, αZEL, and βZEL were conjugated to their respective 14-, and 16-glucosides at differing ratios. The highest rate of 16-glucoside formation was observed with βZEL, the lowest with ZEN. In all cases, 14,16-di-glucoside synthesis from the mono-glucosides occurred relatively slow compared to initial mono-glucoside formation. When ZEN-16-G was incubated with UDP-glucose in order to determine the formation rate of ZEN-14,16-diG, the formation of ZEN-14-G was also observed, indicating reversibility of the glucosylation catalyzed by *Hv*UGT14077. Indeed, ZEN was released by the enzyme when ZEN-14-G and ZEN-16-G (25 µM) were incubated with 10 mM UDP. However, the reverse reaction rates were particularly low with less than 1% compared to initial mono-glucoside synthesis.

Kinetic data were analyzed by the sum of 14-/16-glucoside synthesis rates determined under steady state conditions. The *K*_m_ value for UDP-glucose was determined with 25 µM ZEN and estimated as 78 ± 9 µM (Figure S1). Similar to our previous observations with the DON-conjugating *Os*UGT79 [38], substrate inhibition by ZEN and to lesser extent by the ZELs was indicated (Figure S1). In order to ensure that the experiments satisfied the requirements of steady state kinetic conditions, i.e., to exclude the influence of product inhibition by UDP or the ZEN-glucosides on the obtained results, kinetic assays were conducted such to obtain the lowest possible product formation (e.g., <2% molar in the case of ZEN), that still remained within the quantification limits of the analytical method. Furthermore, the extent of product inhibition was estimated, showing that inhibition by UDP occurred with an IC_50_ of 0.34 mM UDP at 10 mM UDP-glucose and 25 µM ZEN (Figure S1). Therefore, it appears rather unlikely that UDP would have caused a detectable inhibition in these assays. E.g., <2% molar product formation, would imply less than 2 µM UDP at 100 µM ZEN. Product inhibition by ZEN-14-G or ZEN-16-G was not observed when applied at equimolar concentrations with ZEN. The kinetic model of Haldane assuming uncompetitive inhibition of the enzyme-substrate complex by the substrate was thus used for data interpretation. This approach suggested that *Hv*UGT14077 possesses high affinity for ZEN, αZEL, and βZEL with apparent *K*_m_ values in the low micromolar range (Table 2). ZEN clearly is the kinetically favored substrate judged by catalytic efficiency (*k*_cat_/*K*_m_). The highest *k*_cat_ was observed with βZEL. Due to the relatively low reaction rates, formation of the di-glucosides was not observed/detected under steady state conditions. 

*Hv*UGT14077 was also highly active with the flavonoids quercetin and kaempferol. At 3 µM substrate concentration (≈*K*_m_ of ZEN) the specific activities were 100% (kaempferol) and 89% (quercetin) compared to ZEN as determined with the UDP-Glo^TM^ assay. Kinetic analysis with kaempferol did not give clear results as data regression with the Haldane model was not possible. Nevertheless, the obtained results suggest a similar kinetic behavior as with ZEN, with affinity in the same range and rather strong apparent substrate inhibition (Figure S1). In both cases, HRMS analysis revealed the presence of mono-, di-, and tri-glucosides, whereas tetra- and/or penta-glucosides could not be detected. MS/HRMS measurements of the tri-glucoside provided evidence that the flavonoids are conjugated at three different positions since the cleavage of one, two and three glucose moieties was visible (Figures S2 and S3). 

### 2.2. Preparative Synthesis of ZEN-16-Glucoside

**Figure 4:**
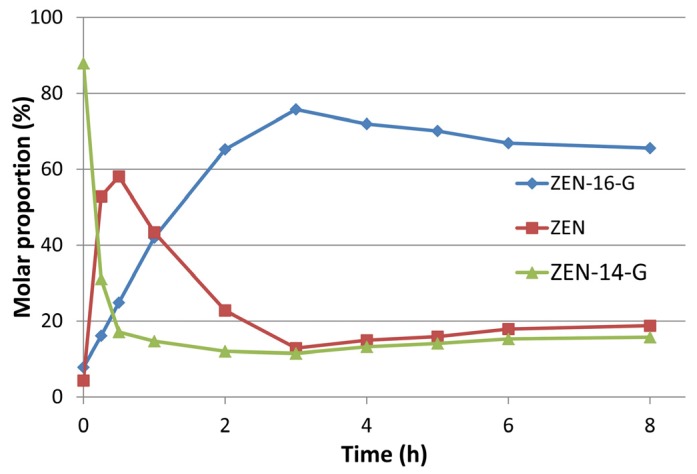
Time course of zearalenone-16-glucoside (ZEN-16-G) synthesis by *Hv*UGT14077, a β-glucosidase from *Lactobacillus brevis* hydrolyzing ZEN-14-glucoside (ZEN-14-G) and sucrose synthase *At*SUS1 for UDP-glucose regeneration. Sucrose was added to 750 mM. Time point “0 h” indicates the initial concentrations in the batch (91% ZEN-14-G, 7.3% ZEN-16-G and 1.3% unconverted ZEN) used for this conversion.

Ten milligrams of ZEN (final batch concentration 3 mM) were glucosylated with *Hv*UGT14077 (2 mg·mL^−1^ protein, 10 mM UDP-glucose). After 4 h (37 °C, pH 7.5), the batch contained 91% ZEN-14-G, 7.3% ZEN-16-G, and 1.3% residual ZEN (molar proportions). In a subsequent step, a β-glucosidase from *L. brevis* recently reported to be highly active with ZEN-14-G, but not with ZEN-16-G (25,000-fold difference in specific activities) [39] was co-incubated with *Hv*UGT14077 aiming to increase the relative proportion of ZEN-16-G. To prevent UDP-glucose depletion, sucrose synthase *At*SUS1 was employed for donor recycling. Using the reaction mixture obtained above as starting point, preliminary experiments were conducted with 100 mM sucrose to fuel UDP-glucose recycling catalyzed by *At*SUS1. By incubating the mixture with *Hv*UGT14077 under donor recycling, but without β-glucosidase, the ratios of ZEN, ZEN-14-G and ZEN-16-G remained virtually constant over an eight-hour period (Figure S4). Addition of the β-glucosidase clearly induced a shift towards higher ZEN-16-G concentrations on cost of ZEN-14-G, accelerated by increasing β-glucosidase dose. The highest molar proportion of ZEN-16-G achieved such was ~65% after 4 h with 24 µg·mL^−1^ β-glucosidase, whereupon slow hydrolysis of ZEN-16-G occurred. Increasing the sucrose concentrations accelerated the reaction and reduced the levels of non-glucosylated ZEN, increasing the yield to a maximum of 74% ZEN-16-G with 500 mM sucrose (Figure S5). A final batch conversion was conducted with 24 µg·mL^−1^ β-glucosidase and 500 mM initial sucrose, increased to 750 mM after 2 h. This resulted in a maximum yield of 75% ZEN-16-G after 3 h (Figure 4). From this batch (10 mg initial ZEN), 6.9 mg ZEN-16-Glc and 2.6 g ZEN-14-G, with purities above 98% as determined by HPLC-UV measurements at 200 nm, were obtained. Furthermore, 1.2 mg of ZEN-14,16-di-G with a purity of 93% were obtained from this batch. 

### 2.3. Structure Elucidation of ZEN-14,16-diG and α/βZEL-14,16-diG

Tentative structure elucidation was performed by LC-MS/HRMS measurements. For all three di-glucosides the formate adduct [M + HCOO]^−^ was more pronounced than the deprotonated ion species and the following masses were detected: ZEN-14,16-diG *m*/*z* 687.2495 (calc. *m*/*z* 687.2506; Δ*m* = 1.6 ppm); αZEL-14,16-diG *m*/*z* 689.2654 (calc. *m*/*z* 689.2662; Δ*m* = 1.2 ppm) and βZEL-14,16-diG *m*/*z* 689.2656 (calc. *m*/*z* 689.2662; Δ*m* = 0.9 ppm). Applying a low collision energy of just 10 eV to the formate adduct resulted in products corresponding to the ZEN-14,16-diG after the cleavage of one (*m*/*z* 479.1912; calc. *m*/*z* 479.1923; Δ*m* = 2.2 ppm; equal to the ZEN-monoglucosides) and two (*m*/*z* 317.1386; calc. *m*/*z* 317.139, equal to ZEN) glucose moieties (Figure S6). This supported the hypothesis that the glucoside moieties are attached at two different positions at the ZEN-structure since, in the case of the ZEN-14-gentiobioside, only the simultaneous loss of two glucoside moieties is visible (see supporting information of [37]). In case of the proposed αZEL-14,16-diG and βZEL-14,16-diG, we observed the equivalent fragments corresponding to the separate cleavage of the two glucose moieties (see Figures S7 and S8). 

Furthermore, the structure of ZEN-14,16-diG was confirmed by means of 1D (^1^H and ^13^C) and 2D (H,H-COSY, H,C-HSQC, and H,C-HMBC) NMR measurements (see Table 3). Using these 2D methods all signals of the ZEN moiety were assigned, and the attachment of two glucose units could unambiguously be proven by long range correlations of their H-1 signals to C-14 and C-16 of ZEN, respectively, in the HMBC spectrum.

## 3. Discussion

One objective of the present study was to provide a functional analysis of *Hv*UGT14077, previously validated as a ZEN-conjugating glucosyltransferase through heterologous expression in yeast. The biochemical analysis of UGTs is of interest to understand the mechanistic principles of glucosylation as involved in plant defense. However, it is clear that *in vitro* characterization alone is a poor foundation to infer the actual physiological function of UGTs as they can be quite promiscuous with regard to their aglycon specificities [15,18]. A prominent example is *At*UGT73C6 from *Arabidopsis* which displays broad substrate diversity [40,41,42] and even conjugates flavonoids that are not natural secondary metabolites of *Arabidopsis* [41]. *At*UGT73C6 was also the first UGT found able to conjugate ZEN, and as judged by competition with the human estrogen receptor, doing so with high affinity [30]. Over-expression of *At*UGT73C6 in *A. thaliana* demonstrated its ability to even glucosylate steroid hormones (brassinosteroids) *in planta* [43]. To narrow down the physiological function of this enzyme just by its functional profile seems pointless as it is able to conjugate substrates as diverse as endogenous secondary metabolites, plant hormones, and exogenous toxins. Several studies reported increased *AtUGT73C6* mRNA levels induced by diverse abiotic and biotic stress factors, including endogenous and exogenous toxic stimuli [14,40,44,45,46,47,48]. It was, thus, proposed that *At*UGT73C6 may act as an unspecific catalyst assisting a mechanism providing broad-range defense [43,45]. 

*Hv*UGT14077 may have a similar function. The gene is up-regulated upon infection of barley with *Fusarium* and by DON application, and a role in defense appears obvious. Based on a phylogeny of UGTs [49], both *At*UGT73C6 (group D1) and *Hv*UGT14077 (group D2) were assigned to group D of family 1 UGTs [32], a clade known to contain UGTs with high substrate diversities [15]. The present study confirms the previous observation that *Hv*UGT14077 is inactive with the trichothecene toxins DON and nivalenol. The enzyme is highly active with ZEN and its phase I metabolites and displays remarkably high affinities to these substrates. This is also indicated by a comparison with the deoxynivalenol-conjugating *Os*UGT79 from rice [38] which was characterized under comparable conditions. While both enzymes present similar turnover numbers (*k*_cat_ of *Os*UGT79 = 0.57 s^−1^ with DON; here, 0.54 s^−1^ with ZEN), we observed a tremendous difference in substrate affinities. The *K*_m_ of *Os*UGT79 with DON is 230 µM, the *K*_m_ of *Hv*UGT14077 with ZEN is 3 µM. The high affinity/catalytic efficiency of *Hv*UGT14077 with ZEN is, therefore, difficult to dismiss as a mechanistic coincidence or mere side activity. Since glycosylation of flavonoids is a trait often observed with several related enzymes of group D [15], we also investigated glucosylation of two representatives, the flavonols kaempferol and quercetin. Interestingly, *Hv*UGT14077 is also highly active with kaempferol and quercetin, apparently with similar affinity/efficiency compared to ZEN. Like ZEN and its phase I metabolites, both flavonoids are presumably conjugated at different positions. This indicates flexibility in substrate accommodation at the aglycon binding site and we propose that similar to *At*UGT73C6, *Hv*UGT14077 may be a rather unspecific UGT recognizing small hydrophobic molecules. 

ZELs are the major phase I metabolites of ZEN and present an additional hydroxyl-group for conjugation at C-7. We were interested to observe whether synthesis of their 7-*O*-glucosides might be catalyzed by *Hv*UGT14077 as well, but formation of such metabolites was not indicated by LC-MS analysis. A βZEL-tri-hexoside was identified in *Arabidopsis* [31], but a corresponding tri-glucoside could not be detected with *Hv*UGT14077. Therefore, and as also suggested by the lower catalytic efficiencies observed with α- and βZEL compared to ZEN, a direct link of *Hv*UGT14077 to phase I activation of ZEN is not evident.

*Hv*UGT14077 also synthesizes the 14,16-di-glucosides of ZEN and αZEL/βZEL, but their low formation rates would suggest that di-glucoside formation is a side activity or even an artifact created in vitro by an artificial excess of UDP-glucose. Whether the formation of such di-glucosides is of biological relevance has yet to be investigated. The availability of suitable analytical standards resulting from this paper provides the means to determine their occurrence in natural samples. 14,16-di-glucoside has previously been described to be formed by the fungus *Thamnidium elegans* [50], but we were unable to reproduce this result with the strain NRRL1613 we obtained from the Agricultural Research Service (ARS) Culture Collection (NRRL). In any case it is much more efficient to generate and purify the glucosides from the enzymatic reaction mix than from the complex culture media of yeast expressing appropriate glucosyltransferases [36] or from fungi with glycosylation ability [51]. The yield and ease of formation also makes the enzymatic synthesis preferable to organic synthesis [52]. *Hv*UGT14077 efficiently converts ZEN to ZEN-14-G, with ZEN-16-G merely occurring as a side product. By applying a bacterial β-glucosidase together with *Hv*UGT14077 and co-substrate regeneration with sucrose/sucrose synthase we could develop a rather unusual, though efficient, procedure to increase the yield of ZEN-16-G. Thus, we are now able to provide sufficient amounts of the scarcely studied metabolite ZEN-16-G for toxicological and metabolic studies.

## 4. Conclusions

This paper reports a functional analysis of *Hv*UGT14077, a defense related UDP-glucosyltransferase possessing high affinities/activities with the mycotoxin ZEN and its phase I metabolites. Furthermore, the capability of *Hv*UGT14077 to efficiently glucosylate two flavonoids (kaempferol/quercetin) and to conjugate its substrates at distinct positions implies flexibility. This led us to conclude that, like previously studied, phylogenetically-related UGTs, *Hv*UGT14077 may serve as a rather unspecific catalyst in vivo. This paper provides a biochemical perspective for further analyses which will be required to obtain deeper insight into the biological function of this enzyme. From a practical point of view, *Hv*UGT14077, recombinantly produced with *E. coli* and one-step affinity purified, proved to be an efficient catalyst suited for the preparative synthesis of several ZEN/ZEL-glucosides. This allows a relatively convenient production of these “masked mycotoxins” in order to provide sufficient amounts for toxicological studies.

## 5. Materials and Methods

### 5.1. Materials

Uridine 5′-diphosphoglucose disodium salt hydrate (UDP-glucose) from *Saccharomyces cerevisiae* (cat. no. U4625) was purchased from Sigma-Aldrich (Vienna, Austria). *Escherichia coli* BL21 (DE3) was from Invitrogen (Carlsbad, CA, USA), *E. coli* Rosetta™ (DE3) from Novagen (Madison, WI, USA). αZEL and βZEL used for enzyme assays were obtained from Sigma-Aldrich (cat. no. Z0166 and Z2000, respectively).

Reverse osmosis water was further purified using a Purelab Ultra system (ELGA, LabWater, Celle, Germany). Methanol (LC-MS grade) and ammonium acetate were obtained from Sigma-Aldrich. The analytical standards of ZEN (100.1 mg·L^−1^ in acetonitrile), as well as αZEL and βZEL (10.4 and 10.1 mg·L^−1^ in acetonitrile, respectively), were purchased from Romer Labs (Tulln, Austria). The standards of the ZEN- and ZEL-glucosides were enzymatically produced as described below in the respective sections from zearalenone previously obtained according to the following procedure. *Fusarium graminearum* strain 02-264 (WG-9, NRRL 66037) [53] was sporulated in mung bean broth (10 g mung beans per liter of water). Solid rice medium was prepared for the cultivation of the strain by adding 10 g rice and 10 mL distilled water to each of the 45 jars (200 mL) and autoclaving them before usage. To each jar 25,000 conidiospores were added and incubation was performed at 27 °C in a dark/light cycle for 24 days. Each jar was extracted with 80 mL ethyl acetate first by using an Ultra-Turrax T25 from IKA-Werke (Staufen, Germany) and then for 90 min at room temperature on a GFL rotary shaker (Burgwedel, Germany). The extracts of all glasses were combined and evaporated to dryness on silica gel. Thereafter, purification was performed on a self-packed silica gel column (40 mm × 800 mm, 63–200 µm, Sigma-Aldrich, Vienna, Austria). The column was packed and washed with petroleum ether and ZEN was eluted from the column by a mixture of petroleum ether and ethyl acetate (3:1, *v*:*v*). The eluate was evaporated to dryness and slightly yellow ZEN-crystals (4.3 g from 450 g rice) with a purity of >90% (determined by HPLC-UV measurements at 200 nm) were obtained. 

### 5.2. Cloning

The *HvUGT14077* gene (GenBank accession GU170356.1) was amplified using the forward primer 5′-GATATACATATGGCTGTCCACGACG-3′ (*Nde*I) and the reverse primer 5-TATATAAAGCTTGCTGGCCTGGATGTCTTC-3′ (*Hin*dIII). The amplified fragment was cloned into plasmid pCA02, a derivative of pKLD116 [54] (Figure S9) using the respective restriction sites underlined on primers. This construct encodes a fusion protein with an *N*-terminal His_6_-tag and maltose binding protein (MalE), followed by a tobacco etch virus (TEV) protease cleavage site, and the C-terminal target gene. The resulting gene product (nHis6-MalE-TEV-*Hv*UGT14077) has a predicted molecular mass of 97 kDa and was expressed with *E. coli* BL21 (DE3). 

Sucrose synthase (*At*SUS1) from *Arabidopsis thaliana* (TAIR accession AT5G20830.1; GenBank accession BAH19538.1) was expressed in the same topology (nHis_6_-MalE-TEV-*At*SUS1, pCA02) with *E. coli* Rosetta 2.

### 5.3. Protein Expression and Purification

Recombinant protein production (*Hv*UGT14077 and *At*SUS1) was carried out in terrific broth (tryptone 10 g·L^−1^, yeast extract 20 g·L^−1^, glycerol 5 g·L^−1^, K_2_HPO_4_·3H_2_O 14 g·L^−1^, KH_2_PO_4_ 5.2 g·L^−1^) with 100 mg·L^−1^ ampicillin, additionally supplemented with 35 mg·L^−1^ chloramphenicol for *E. coli* Rosetta 2. Isopropyl-β-D-1-thiogalactopyranoside (IPTG, 1 mM final concentration) was added when the optical density (OD_600_) reached 0.5. The flasks were further incubated for 20 h at 20 °C and 100 rpm. After that period, the biomass was harvested by centrifugation (3300× *g*, 30 min) and re-suspended in 25 mM Tris/Cl (pH 7.5) + 500 mM NaCl/25 mM imidazole, the binding buffer for the first purification step. The cells were disrupted by sonication with a Branson Sonifier W-250 D (Branson Ultrasonics Corporation, Danbury, CT, USA). The cell extract was cleared by centrifugation at 40,000× *g*. Protein purification was performed by IMAC on Ni^2+^-charged HisTrap Crude FF columns, 5 mL (GE Healthcare, Vienna, Austria). The target protein was bound to the column in the above-specified binding buffer. Protein was eluted with the same buffer containing 500 mM imidazole. After IMAC, the buffer was changed to 50 mM potassium phosphate (pH 7) + 50 mM NaCl/10% (*w*/*v*) glycerol by size exclusion chromatography on Sephadex G25 (GE Healthcare). *Hv*UGT14077 and *At*SUS were stored at −80 °C. 

Protein concentrations were determined with the Bio-Rad (Vienna, Austria) protein assay based on the dye-binding method of Bradford. Bovine serum albumin (Sigma-Aldrich P5619) was used as standard. Sodium dodecyl sulfate polyacrylamide gel electrophoresis (SDS-PAGE) including Coomassie blue staining was done with a 12% separation and a 7% stacking gel. 

### 5.4. Glycosylation Assays

All enzyme assays were conducted in 100 mM Tris/Cl (pH 7.5) at 37 °C with 2–4 min reaction time. *Hv*UGT14077 was added in concentrations of 0.1–2 mg·mL^−1^. The substrate concentrations of ZEN, α/βZEL were 1–100 µM for kinetic assays, UDP-glucose was added to a concentration of 10 mM. To determine the *K*_m_ value for UDP-glucose, 25 µM ZEN were incubated with varying concentrations of UDP-glucose (0.02–10 mM). Inhibition by UDP (0.05–10 mM) was determined with 10 mM UDP-glucose. 

The assays were stopped by transferring 20 µL of sample to 180 µL methanol. After centrifugation (20,000× *g*, 5 min) to remove precipitated protein, the samples were further diluted in methanol (ZEN) or H_2_O (α/βZEL) to an expected analyte concentration in the range of 0.2–1 mg·L^−1^. The concentrations of ZEN, αZEL, βZEL and their glucosides were determined by LC-MS/MS as described below. Enzyme activity is reported as nmol·min^−1^·mg^−1^ or μmol·min^−1^·mg^−1^ referring to glucoside formation per mg of protein as determined with the Bradford method. Data analysis for kinetic enzyme characterization was performed with SigmaPlot 11.0 (Systat Software, San Jose, CA, USA).

Assays with kaempferol and quercetin were conducted in 100 mM Tris/Cl (pH 7.5) at 37 °C and 1 mM UDP-glucose. Enzyme activity was determined by quantifying released UDP with the UDP-Glo^TM^ glycosyltransferase assay from Promega (Madison, WI, USA).

### 5.5. Batch Glycosylation and ZEN-16-G Production

Preparative synthesis of ZEN-glucosides was performed at pH 7.5 (100 mM Tris/Cl) at 37 °C. Due to the low water solubility of ZEN (ca. 10 mg·L^−1^) [55] it was dissolved in dimethyl sulfoxide (DMSO) to 10 g·L^−1^ and added stepwise by increasing its concentration by 200 mg·L^−1^ each 30 min, resulting in final concentrations of 10% DMSO and 1 g·L^−1^ ZEN (3 mM) in the batch reaction. *Hv*UGT14077 was added to 2 mg·mL^−1^ with 10 mM UDP-glucose. After 4 h, the batch was frozen at −20 °C and stored until LC-MS/MS results were available. Afterwards, this batch was used to biocatalytically increase the ZEN-16-G proportion (sum of ZEN/ZEN-glucosides 1.5 mM in reaction), by adding a β-glucosidase from *Lactobacillus brevis* [56] with 0.8–24 µg·mL^−1^ in preliminary assays and 24 µg·mL^−1^ in a final batch. Fresh *Hv*UGT14077 and *At*SUS1 for UDP-glucose recycling were added to final concentrations of 1.25 mg·mL^−1^ each. Sucrose, the substrate for *At*SUS1 was added to concentrations of 100–500 mM in preliminary tests and 750 mM in the final batch reaction. 

### 5.6. LC-MS/MS Analysis

A 1290 ultra-high-performance liquid chromatography system (UHPLC) from Agilent Technologies (Waldbronn, Germany) equipped with a Kinetex core-shell C18 column (150 mm × 2.1 mm, 2.6 µm; Phenomenex, Aschaffenburg, Germany) was used for chromatographic separation at a temperature of 30 °C. The eluents were composed of water and methanol (A: 80:20, *v:v*; B: 3:97, *v:v*) and contained both 5 mM ammonium acetate. The flow rate was set to 250 µL·min^−1^ and the default injection volume was 5 µL. The following gradient was applied: The starting condition of 10% B was held for 0.1 min, following by a linear increase to 100% B at 9 min. After a holding time of 2.9 min, a fast switch back to the starting conditions was performed within 0.1 min and the column was equilibrated for 3 min. Between 2 and 9.5 min the LC flow was directed to the MS. 

Mass spectrometric detection was performed on a QTrap 4000 (Sciex, Foster City, CA, USA) and the source conditions were as follows: curtain gas at 30 psi (207 kPa), ion spray voltage (−4.2 kV), temperature of 550 °C, ion source gas 1 and 2, both at 50 psi (344 kPa). Starting with proposed selected reaction monitoring (SRM) transitions, the target compounds were optimized when the standards became available. The following SRM transitions were used with a dwell time of 25 ms: ZEN (retention time 8.2 min) *m*/*z* 317.1 (declustering potential, DP, −90 V), product ions *m*/*z* 131.0 (collision energy, CE, −42 V) and *m*/*z* 175.0 (CE, −34 V). αZEL (8.1 min) and βZEL (7.5 min) were both measured by *m*/*z* 319.1 (DP, −95 V), product ions *m*/*z* 275.1 (CE, −30 V) and *m*/*z* 160 (CE, −42 V). ZEN-14-G (6.5 min), and ZEN-16-G (5.3 min) had the same quantifier transition *m*/*z* 479.1 (DP, −95 V) to *m*/*z* 317.0 (CE, −22 V), but the qualifier product ion was *m*/*z* 175.0 (CE; −54 V) for ZEN-14-G and *m*/*z* 149.0 (−54 V) for ZEN-16-G. αZEL-14-G (6.5 min), βZEL-14-G (5.6 min), αZEL-16-G (5.1 min), and βZEL-16-G (4.7 min) were all measured with the following transitions *m*/*z* 481.1 (DP, −100 V), product ions *m*/*z* 319.0 (CE, −18 V), and *m*/*z* 275.0 (CE, −48 V). ZEN-14,16-diG (4.2 min) was the only compound with an acetate adduct as precursor after optimization *m*/*z* 701.1 (DP, −75 V), product ions *m*/*z* 317.1 (CE, −18 V) and *m*/*z* 275.0 (CE, −48 V). For αZEL- (3.9 min) and βZEL-diG (3.6 min) the following proposed (non-optimized transitions) were used *m*/*z* 643.2 (DP, −100 V), product ions *m*/*z* 319.1 (CE, −40 V) and *m*/*z* 481.1 (CE, −20 V). 

### 5.7. ZEN-16-G Preparative Purification

For the purification of the formed ZEN-metabolites an 1100 series preparative HPLC from Agilent Technologies equipped with a Gemini NX C18 column (150 mm × 21.2 mm; 5 µm; Phenomenex) was used. The eluents were water (eluent A) and methanol (eluent B) and the following gradient with a flow rate of 16 mL·min^−1^ was applied: 40% B were held for 1 min and then the percentage of B was linearly increased to 100% up to 10 min. The column was flushed with 100% B for 3 min before switching back to the initial conditions within 0.1 min and column equilibration. The default injection volume was 900 µL and the peaks were collected by peak based fractionation (up and down slope 10 units·s^−1^, max. peak duration 0.5 min) based on the UV signal at 270 nm between 4 and 10.5 min. ZEN-14,16-diG eluted at 4.7 min, ZEN-16-G at 6.1 min, ZEN-14-G at 7.5 min and ZEN at 9.7 min. 

### 5.8. αZEL/βZEL-14/16-G Production and Purification

The productions of the ZEL-glucosides were performed according to [57]. Briefly, approximately 3 mg ZEN-14-G or ZEN-16-G were dissolved in 3 mL methanol and the tenfold molar amount of sodium borohydride (ca. 3 mg) dissolved in 0.5 mL methanol were immediately added. The solution was vortexed from time to time and after ca. 30 min the completion of the reaction was confirmed by LC-MS/MS measurements and stopped by the addition of 30 µL acetic acid. The resulting racemic mixture of the αZEL- and βZEL-form of the glucosides was purified using the preparative HPLC system. The eluents were water (eluent A) and methanol (eluent B) and the flow rate was set to 16 mL·min^−1^. For the purification of the ZEL-glucosides a SunFire prep C18 OBD column (100 mm × 19 mm, 5 µm, Waters Corporation, Milford, MA, USA) was used and the compounds were collected by peak-based fractionation at 270 nm. In case of αZEL-14-G and βZEL-14-G the peak based fractionation was set to 1 unit·s^−1^ for up and down slope and a maximum peak duration of 0.4 min. The applied gradient was as follows: 0–1.0 min: 40% B, 1.0–7.0 min linear increase from 40% to 80% B, 7.0–7.1 min fast switch to 100% B and holding until 10 min. Thereafter, a fast switch within 0.1 min to the initial conditions was performed before equilibrating the column. αZEL-14-G eluted at 6.3 min and βZEL-14-G at 5.1 min. In case of αZEL-16-G and βZEL-16-G the peak-based fractionation was set to up slope 3 unit·s^−1^ and down slope 1 unit·s^−1^ and a max. peak duration of 0.6 min. The gradient was adapted as follows: 0–1.0 min: 35% B, 1.0–7.0 min linear increase from 35% to 60% B, 7.0–7.1 min fast switch to 100% B and holding until 10 min. Then the starting conditions were reached within 0.1 min and the column was equilibrated. αZEL-16-G eluted at 6.9 min and βZEL-16-G at 5.2 min. 

### 5.9. Characterization of ZEN-14,16-diG, αZEL-14,16-diG, and βZEL-14,16-diG

MS/HRMS measurements were performed using a 1290 UHPLC system connected to a 6550 iFunnel quadrupole time of flight system (both from Agilent Technologies). A Zorbax SB-C18 (150 mm × 2.1 mm, 1.8 µm) column was used and the eluents consisted of water (eluent A) and methanol (eluent B) and contained both 0.1% formic acid and 1 mM ammonium formate. The following gradient was applied: 10% B were held for 0.5 min and then linearly increased to 100% B at 20 min. The column was flushed for 2 min before switching back to the initial conditions within 0.1 min and equilibrating the column until the end of the chromatographic run (25 min). The source conditions were as follows: gas temperature 130 °C, drying gas flow 14 L·min^−1^, sheath gas temperature 300 °C and flow 10 L·min^−1^. The nebulizer was set to 30 psig and the capillary and nozzle voltage were 4 kV and 0.5 kV, respectively. MS spectra were acquired between *m*/*z* 100 to 1000 with a scan rate of three spectra per seconds. In MS/MS mode the same scan rate was used, but spectra were acquired between *m*/*z* 50–700 and the isolation width was set to an *m*/*z* of 1.3. Quercetin and kaempferol measurements were performed with the same method.

### 5.10. Confirmation of ZEN-14,16-diG Structure by NMR

NMR spectra were obtained in methanol-d_4_ at 293 K on a Bruker Avance III HD spectrometer (Bruker BioSpin GmbH, Rheinstetten, Germany) equipped with a 5-mm Cryoprobe™ Prodigy BBO, operating at 600.15 MHz for ^1^H and 150.90 MHz for ^13^C. NMR data were recorded and evaluated using TopSpin 3.2 (Bruker BioSpin GmbH). Chemical shifts were established based on residual solvent signals (3.31 ppm for ^1^H and 49.15 ppm for ^13^C) and reported relative to tetramethylsilane.

## Figures and Tables

**Table 1:** Reaction rates of *Hv*UGT14077 at pH 7.5 (100 mM Tris/Cl) and 37 °C with 25 µM substrate and 10 mM UDP-glucose or UDP in case of the reverse reaction. “Z” indicates the aglycons zearalenone (ZEN), α-zearalenol (αZEL), or β-zearalenol (βZEL). The results are the means of triplicate determination ± standard deviations.

Reaction	Reaction Rate (nmol·min^−1^·mg^−1^)		
	ZEN	αZEL	βZEL
Z → Z-14-G ^1^	213 ± 14	176 ± 7	351 ± 20
Z → Z-16-G ^1^	45 ± 1	61 ± 7	227 ± 19
Z-14-G → Z-14,16-diG ^2^	0.64 ± 0.13	0.47 ± 0.13	0.82 ± 0.14
Z-16-G → Z-14,16-diG ^2^	1.6 ± 0.1	2.6 ± 0.1	1.6 ± 0.1
Z-14-G → Z ^3^	0.55 ± 0.01	0.32 ± < 0.01	0.75 ± 0.01
Z-16-G → Z ^3^	1.1 ± < 0.1	0.59 ± 0.01	0.93 ± 0.02

^1^ ZEN, α/βZEL incubated with 10 mM UDP-glucose; ^2^ mono-glucosides of ZEN, α/βZEL incubated with 10 mM UDP-glucose; ^3^ mono-glucosides of ZEN, α/βZEL incubated with 10 mM UDP.

**Table 2:** Kinetic constants of recombinant *Hv*UGT14077 determined at 37 °C and pH 7.5 (100 mM Tris/Cl). The displayed values are the means of three independent replicates ± standard deviations. Reaction rates (*V*_max_/*k*_cat_) refer to the sum of respective 14- and 16-glucosides.

Substrate	Kinetic Constant			
	*K*_m_ (µM)	*V*_max_ (µmol·min^−1^·mg^−1^)	*k*_cat_ (s^−1^)	*k*_cat_/*K*_m_ (s^−1^·mM^−1^)
Zearalenone	3 ± 1	0.34 ± 0.06	0.54 ± 0.10	190
α-Zearalenol	13 ± 2	0.32 ± 0.03	0.52 ± 0.05	40
β-Zearalenol	27 ± 3	1.2 ± 0.1	2.0 ± < 0.1	74

**Table 3:** ^1^H (δ, ppm; multiplicity; J, Hz) and ^13^C (δ, ppm) NMR data of zearalenone-14,16-di-glucoside.

Position	^1^H	^13^C
1	-	169.4
2	1.37 (d, 6.3)	20.3
3	5.29 (m)	73.2
4	1.74 (m); 1.58 (m)	36.0
5	1.78 (m); 1.60 (m)	22.3
6	2.48 (m); 2.28 (m)	44.7
7	-	214.3
8	2.66 (m); 2.24 (m)	38.3
9	2.02 (m); 1.60 (m)	22.7
10	2.33 (m); 2.05 (m)	32.5
11	6.10 (ddd, 15.6, 9.8, 4.5)	135.1
12	6.32 (d, 15.5)	129.6
13	6.97 (d, 2.1)	108.6
14	-	160.7
15	6.90 (d, 2.1)	104.2
16	-	156.6
17	-	119.6
18	-	138.1
1′	4.97 (d, 7.4)	102.1
1″	4.98 (d, 7.3)	102.1
2′-5′; 2″-5″	3.62–3.28 (m)	78.5; 78.4; 78.4; 78.0; 75.0; 74.9; 71.8; 71.6
6′; 6″	3.95–3.88 (m); 3.70–3.62 (m)	62.9; 62.9
6′; 6″	3.95–3.88 (m); 3.70–3.62 (m)	62.9; 62.9

## Supplementary Materials

The following are available online at www.mdpi.com/2072-6651/9/2/58/s1, Figure S1: Kinetic analysis of *Hv*UGT14077, Figure S2: High resolution tandem mass spectrometric product ion scan of the tentatively identified kaempferol-tri-glucoside in positive electrospray ionization mode at a collision energy of 15 eV, Figure S3: High-resolution tandem mass spectrometric product ion scan of the tentatively identified quercetin-tri-glucoside in positive electrospray ionization mode at a collision energy of 15 eV, Figure S4: Synthesis of zearalenone-16-glucoside (ZEN-16-G) with *Hv*UGT14077 (1.25 mg·mL^−1^), different concentrations of a β-glucosidase from *Lactobacillus brevis* and sucrose synthase *At*SUS1, Figure S5: Synthesis of zearalenone-16-glucoside (ZEN-16-G) with *Hv*UGT14077 (1.25 mg·mL^−1^), a β-glucosidase from *Lactobacillus brevis* (24 µg·mL^−1^) and sucrose synthase *At*SUS1 (1.25 mg·mL^−1^) for UDP-glucose regeneration. Sucrose was added in different concentrations, Figure S6: High-resolution tandem mass spectrometric product ion scan of zearalenone-14,16-di-glucoside in negative electrospray ionization mode at a collision energy of 10 eV, Figure S7: High-resolution tandem mass spectrometric product ion scan of the tentatively identified α-zearalenol-14,16-di-glucoside in negative electrospray ionization mode at a collision energy of 10 eV, Figure S8: High-resolution tandem mass spectrometric product ion scan of the tentatively identified β-zearalenol-14,16-di-glucoside in negative electrospray ionization mode at a collision energy of 10 eV, Figure S9: Topology of pCA02.

## Abbreviations

DMSO	Dimethyl sulfoxide
DON	Deoxynivalenol
GT	Glycosyltransferase
HPLC	High performance liquid chromatography
IMAC	Immobilized metal ion chromatography
IPTG	Isopropyl-β-d-1-thiogalactopyranoside
LC-MS	Liquid chromatography coupled to mass spectrometry
LC-MS/MS	Liquid chromatography coupled to tandem mass spectrometry
MS/HRMS	High resolution tandem mass spectrometry
NMR	Nuclear magnetic resonance spectroscopy
UDP	Uridine diphosphate
UGT	Uridine diphosphate (UDP) glycosyltransferase
UHPLC	Ultra high performance liquid chromatography
PSPG	Plant secondary product glycosyltransferase
SDS-PAGE	Sodium dodecyl sulfate polyacrylamide electrophoresis
SRM	Selected reaction monitoring
TEV	Tobacco etch virus
QTL	Quantitative trait locus
αZEL	α-Zearalenol
βZEL	β-Zearalenol
ZEN	Zearalenone
ZEN-14-G	Zearalenone-14-*O*-glucoside
ZEN-14,16-diG	Zearalenone-14,16-diglucoside
ZEN-16-G	Zearalenone-16-*O*-glucoside
