# Supplementary material for: Synthesis of Mono- and Di-Glucosides of Zearalenone and α-/β-Zearalenol by Recombinant Barley Glucosyltransferase HvUGT14077
Source: Toxins (Basel). 2017 Feb 9;9(2):58. doi: 10.3390/toxins9020058 (PMC5331437; doi:10.3390/toxins9020058)
Supplement: Supplementary file 1 [file toxins-09-00058-s001.pdf]

Supplementary Materials: Synthesis of Mono- and Di-Glucosides of Zearalenone and α-/β-Zearalenol by Recombinant Barley Glucosyltransferase *Hv*UGT14077

Herbert Michlmayr, Elisabeth Varga, Francesca Lupi, Alexandra Malachová, Christian Hametner, Franz Berthiller and Gerhard Adam

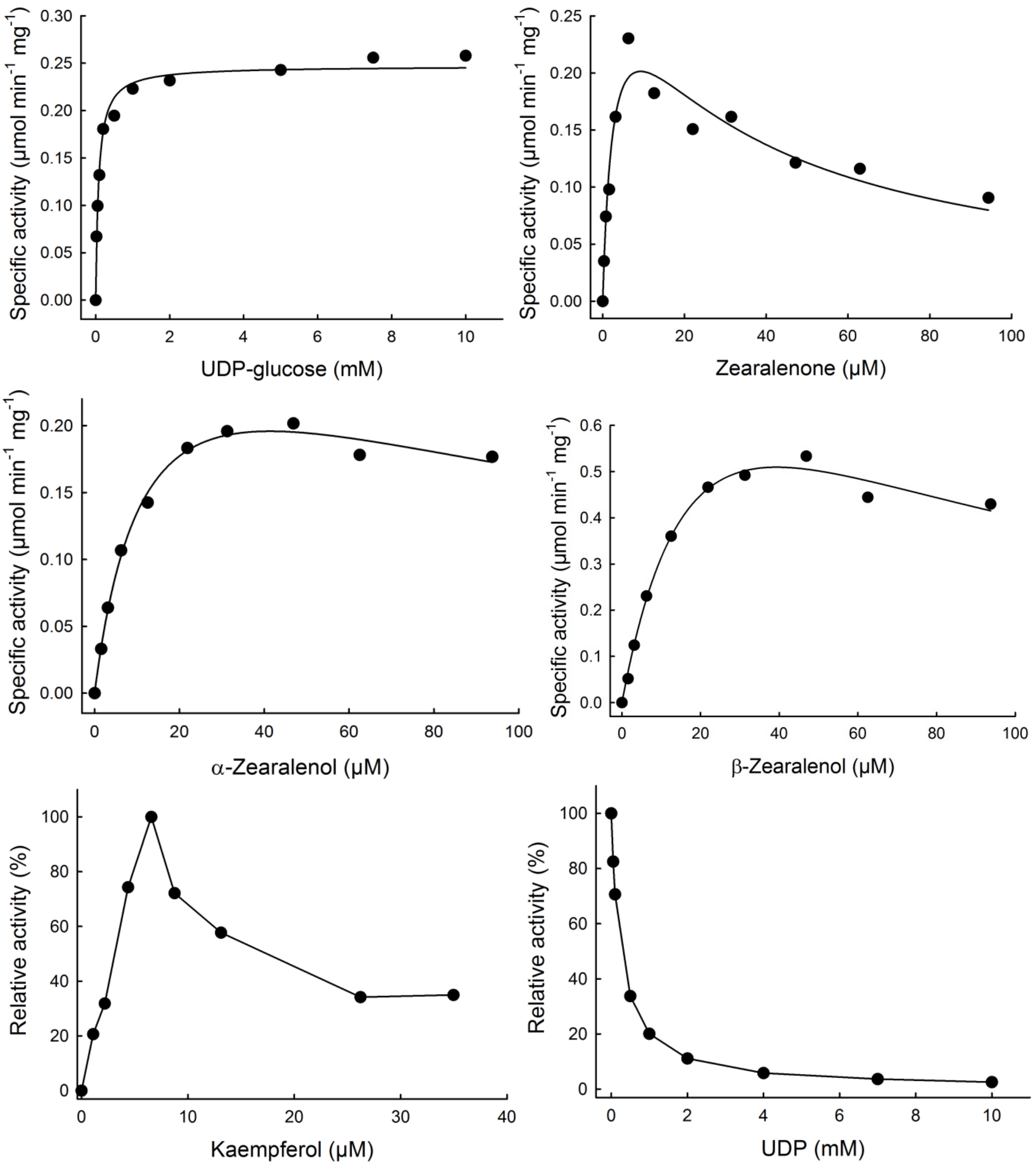

Figure S1. Kinetic analysis of *Hv*UGT14077. All assays were performed at 37 °C, 100 mM Tris/Cl pH 7.5. Kinetic assays with UDP-glucose were done with 25 µM zearalenone. Saturation curves with zearalenone, α-zearalenol and β-zearalenol were determined with 10 mM UDP-glucose. Product formation was quantified by LC-MS/MS. Activity with kaempferol was determined with the UDP-Glo assay from Promega with 1 mM UDP-glucose. Since data regression with the Haldane model was not possible, no fitted curve is displayed in this case. Inhibition by UDP was determined at 10 mM UDP-glucose and 25 µM zearalenone.

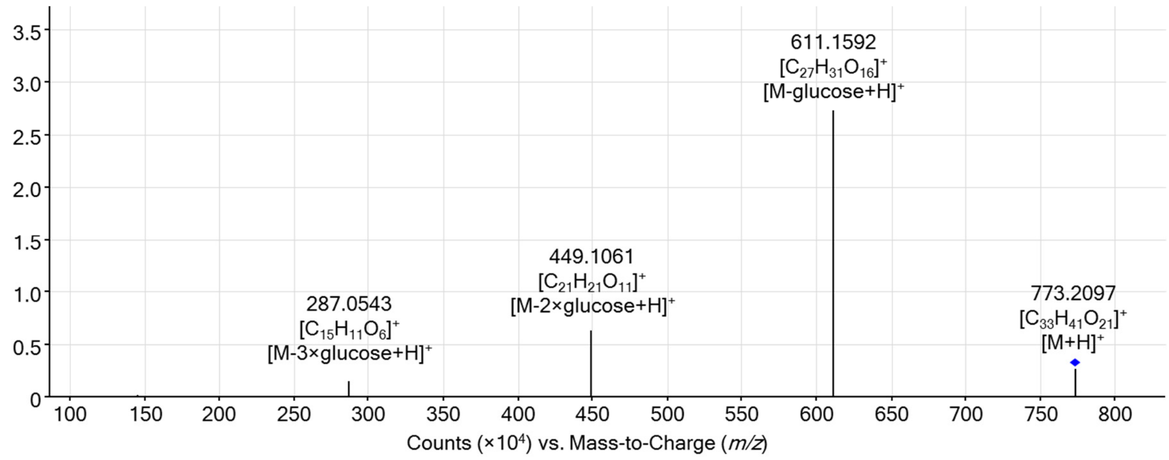

Figure S2. High resolution tandem mass spectrometric product ion scan of the tentatively identified kaempferol-tri-glucoside in positive electrospray ionization mode at a collision energy of 15 eV.

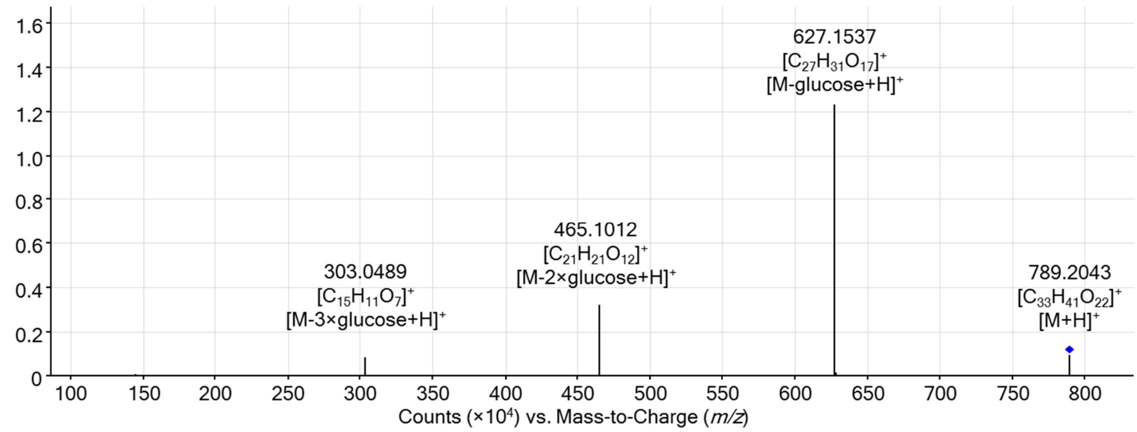

Figure S3. High resolution tandem mass spectrometric product ion scan of the tentatively identified quercetin-tri-glucoside in positive electrospray ionization mode at a collision energy of 15 eV.

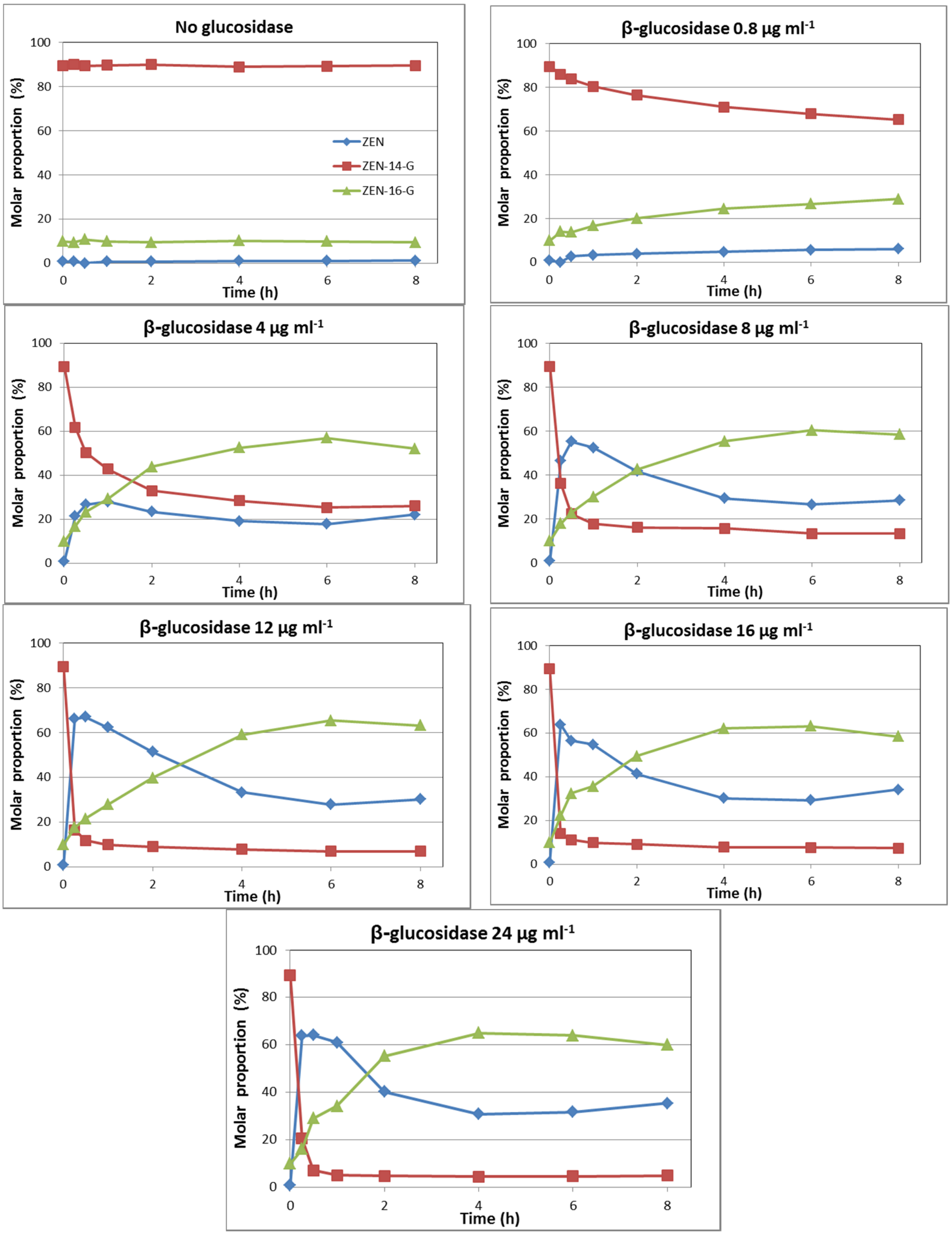

Figure S4. Synthesis of zearalenone-16-glucoside (ZEN-16-G) with *Hv*UGT14077 (1.25 mg·mL⁻¹), different concentrations of a β-glucosidase from *Lactobacillus brevis* and sucrose synthase *At*SUS1 (1.25 mg·mL⁻¹) for UDP-glucose regeneration. Sucrose was added to 100 mM. Time point "0 h" indicates the initial concentrations in the batch (91% zearalenone-14-glucoside, ZEN-14-G; 7.3% ZEN-16-G and 1.3% unconverted zearalenone, ZEN) used for this conversion.

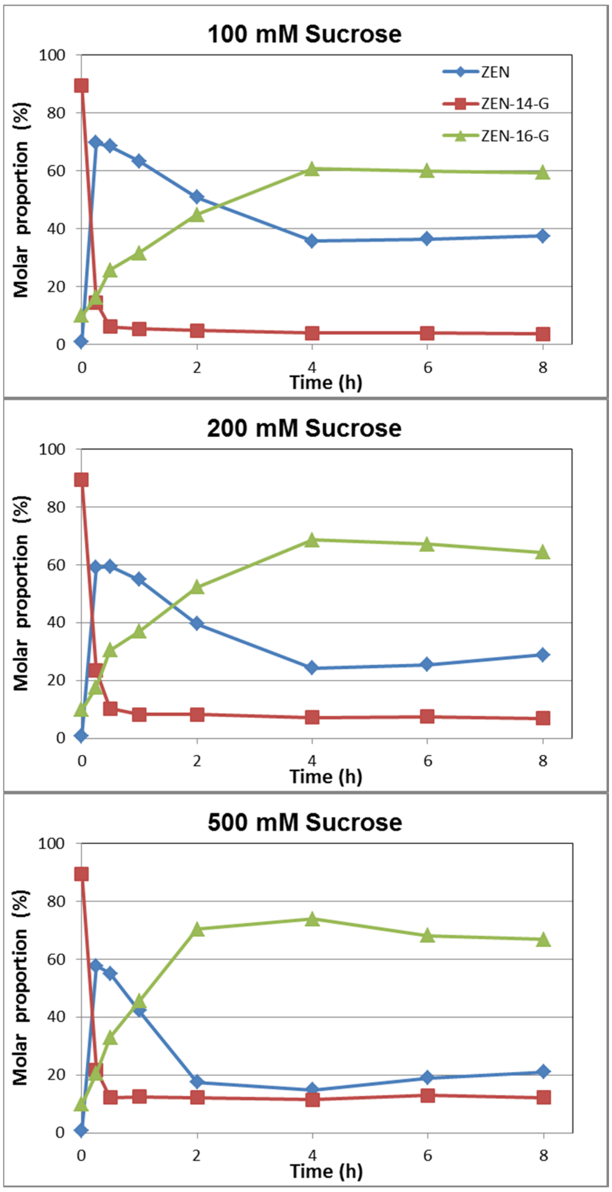

Figure S5. Synthesis of zearalenol-16-glucoside (ZEN-16-G) with *Hv*UGT14077 (1.25 mg·mL^{-1}), a β-glucosidase from *Lactobacillus brevis* (24 µg·mL^{-1}) and sucrose synthase *At*SUS1 (1.25 mg·mL^{-1}) for UDP-glucose regeneration. Sucrose was added in different concentrations. Time point "0 h" indicates the initial concentrations in the batch (91% zearalenone-14-glucoside, ZEN-14-G; 7.3% ZEN-16-G and 1.3% unconverted ZEN) used for this conversion.

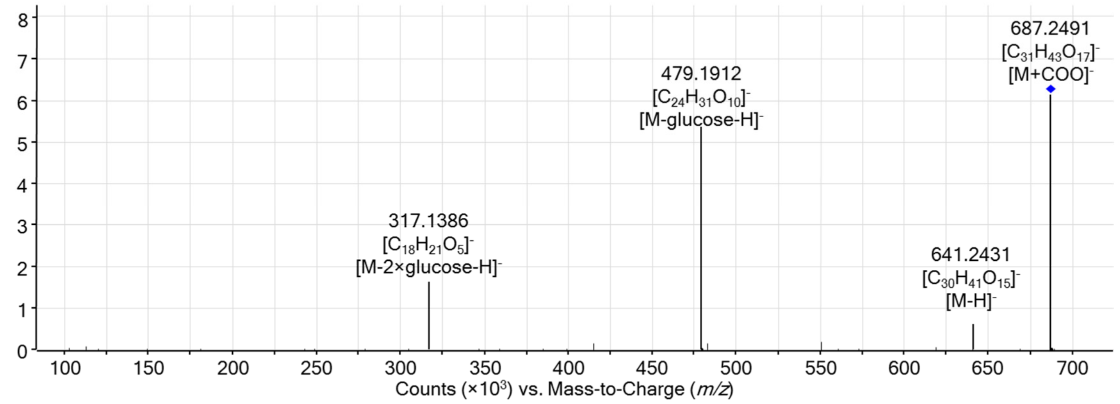

Figure S6. High resolution tandem mass spectrometric product ion scan of zearalenone-14,16-di-glucoside in negative electrospray ionization mode at a collision energy of 10 eV.

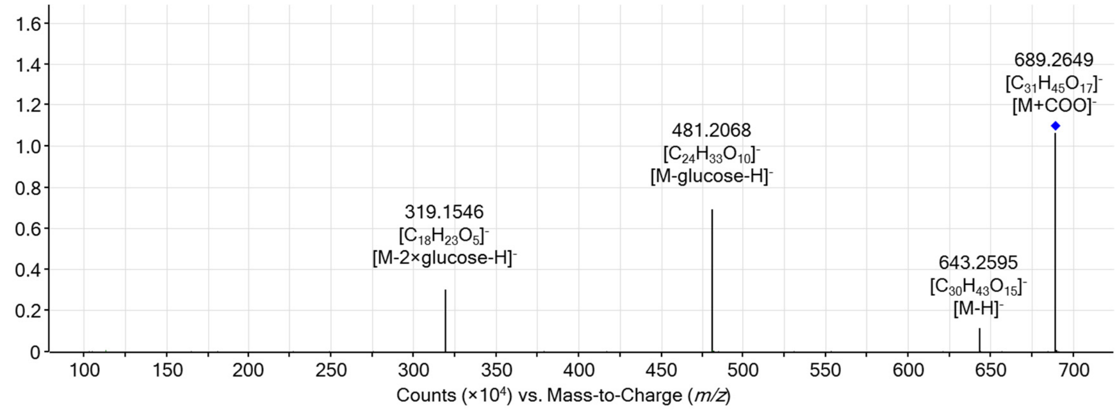

Figure S7. High resolution tandem mass spectrometric product ion scan of the tentatively identified α-zearalenol-14,16-di-glucoside in negative electrospray ionization mode at a collision energy of 10 eV.

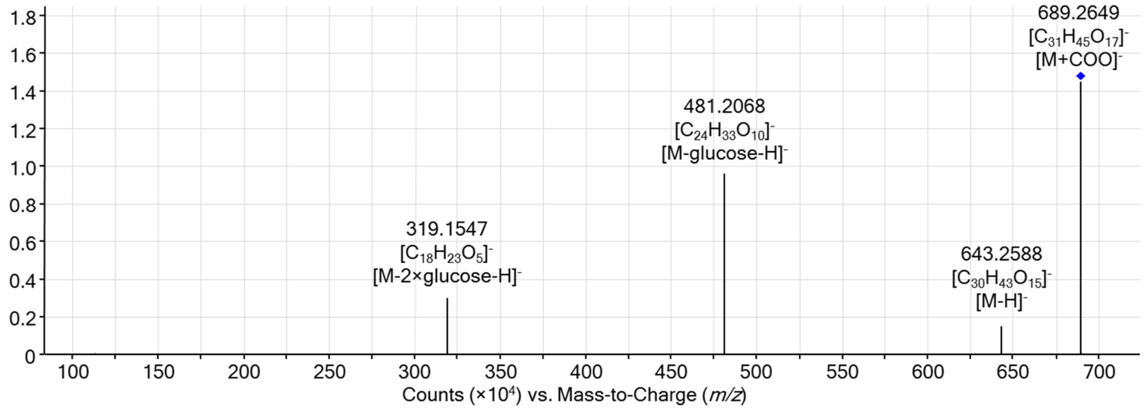

Figure S8. High resolution tandem mass spectrometric product ion scan of the tentatively identified β-zearalenol-14,16-di-glucoside in negative electrospray ionization mode at a collision energy of 10 eV.

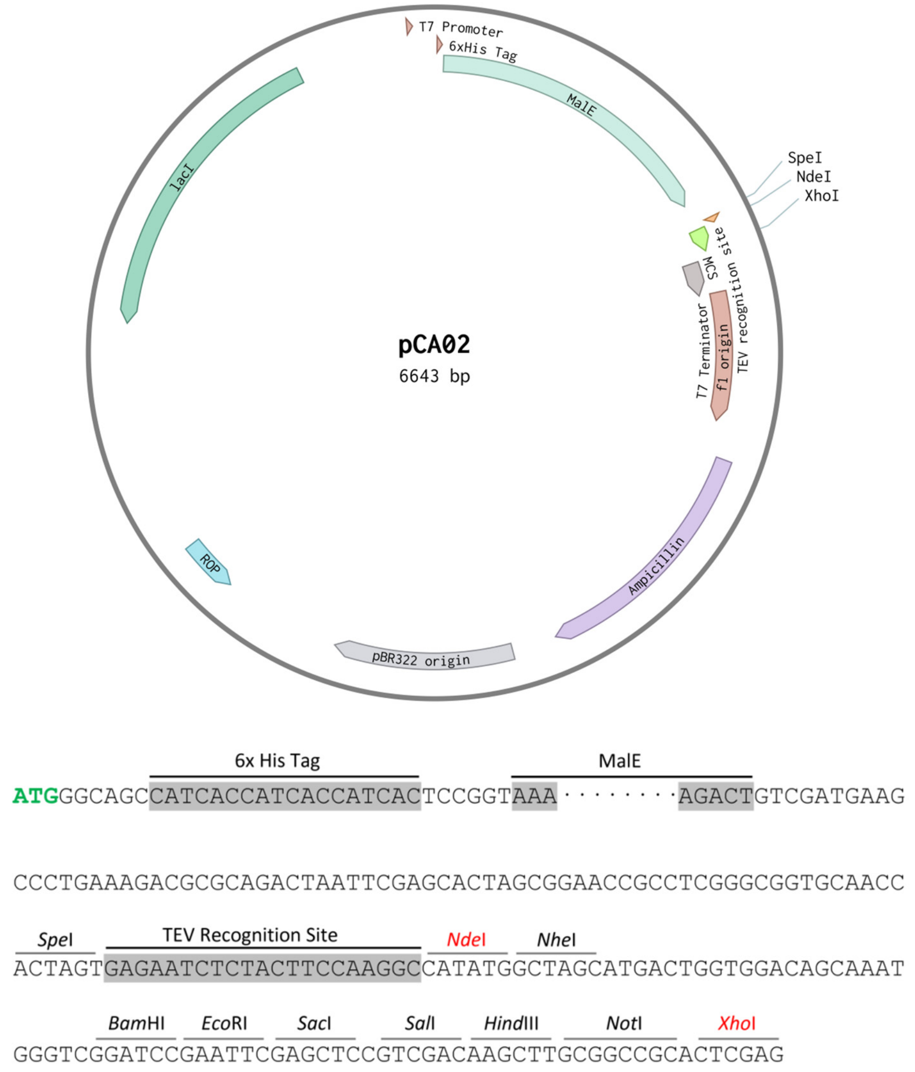

Figure S9. Topology of pCA02. pCA02 is a derivative of pKLD116 [1] which in turn is a derivative of pET21a. pCA02/pKLD116 allow expression of fusion proteins with *N*-terminal His_6-tag, maltose binding protein (MalE gene), TEV recognition site and the *C*-terminal target protein. pCA02 contains the multiple cloning site of the pET21 vector series. The plasmid map was created with Benchling (https://benchling.com/).

References

1. Rocco, C.; Dennison, K.; Klenchin, V.A.; Rayment, I.; Escalante-Semerena, J. Construction and use of new cloning vectors for the rapid isolation of recombinant proteins from *Escherichia coli*. *Plasmid* **2008**, *59*, 231–237.

© 2017 by the authors; licensee MDPI, Basel, Switzerland. This article is an open access article distributed under the terms and conditions of the Creative Commons Attribution (CC BY) license (http://creativecommons.org/licenses/by/4.0/).
